# Dr. Bellamy's Case of Diseased Bladder

**Published:** 1806-08

**Authors:** G. Bellamy

**Affiliations:** His Majesty's Ship Glory, Torbay


### *To the Editors of the Medical and Physical Journal.*

Gentlemen,

I Request you to publish, for the criticism as well as the information of the medical public, a case of diseased bladder, with fistula in perinæo, &c.

I am yours, &c.

G. BELLAMY, M. D.

His Majesty's Ship Glory, Torbay, Feb. 15, 1806.

---

Mr. Andrew Sharp, aged about 38, came under my care the 22d Jan. 1806, for an affection in perinæo; a considerable hardness to be felt, and some enlargement to be seen at the root of the penis, or rather at the bulb of the urethra; feels much pain when touched, and a good deal of internal distress, with sense of fulness and aching, even when the parts are at rest; a great deal more when he evacuates urine or stools; the former runs off in a small narrow stream; does not stop, but requires a little time to begin. An old complaint, was under my care about six months ago, then relieved by small mercurial frictions on the part; is the effect believed of an old gonorrhœa some years ago, and by quacking with improper injections; occasionally a little gleet; looks ill with it; there is no redness, or heat, or sign of inflammation; but supposed to be a chronic thickening, or enlargement: probably the prostate gland is diseased. Utatr. ung. hydrarg. gr. v. bis in die part. affect. heri haust. cath. fotus com. et rep. p. r. n.

23d, Swelling undoubtedly larger, yet not hot or red, but more extensive, and very apparent, with great sense of fullness; pain in making water, but not interrupted, except being a small stream, as is always the case with him, more or less; the physic acted well. ℞. Fotus, & ung. hydrarg. Note, Should not wonder to see foundation of fistula in perinæo.

24th, I am sorry to say, notwithstanding my attempt to promote absorption, that the tumour increases very much, and even with a degree of inflammation, great pain, yet no re-action of the circulation; looks, and feels weak, pale, &c.; still shall persevere to repel, for fear of fistula. ℞. Ung. hydrarg: fot. &c.

25th, Tumour still large, but not more so, nor pointed, but very painful all night, and extremely sensible to the touch; touch; feels most relief by fomentation. I rather hope it will break internally, and thus save an external wound or fistula in perinæo; yet even this may cause destruction, and find its way through; but on the whole better so than fistula in pernæo. A little oozing from the urethra, and more so when pressure is made on the swelling, and carried along that canal: seems to be from the abscess; if so, and it is like matter, may expect ease at least; has had no rigours. ℞. Ung. fotus. &c.

26th, Abscess is very large, nearly as large as a fist, and extending quite to the anus, and on its sides, a little thence up the perinæum, about two inches; will probably contain more than a pint of matter; begins to fluctuate, feels soft and pointing about the left side of the rapha perinæi, an inch and a half from the anus. Great deal of pain on being touched, a little ichorous-like or gleetish matter from penis, rather less than yesterday; the hopes of its bursting inside appear to be done away, and less sign of tracing any flow of matter from the urethra, by pressure on the abscess; strange he has no rigour, but has considerable fever; not great, or any reaction of heat, or fullness of pulse: it is rather quick, face pale and fallen, chiefly from pain; does not appear to be aware of the seriousness of his disease; not yet explained to him; bears it very well; does not groan; lies pretty quiet: got him into the sick birth, where much quieter, cleaner, and comfortable. Keep parts very clean, and him rather low; now must promote suppuration; no hopes of absorption. Appr. cataplasm emoll. ter die cũm fot. com. &c. This will become a very serious case.

27th, The abscess broke last evening, a day or two sooner than I expected; in the morning there was a little vesication, and oozing of serum, which appeared to be the effect of the heat of the poultice; he experienced instant ease; there was great fætor; a considerable spot of the integuments of the abscess broke down, and more about to yield, in form of a blackish-brown eschar; the opening will now admit the little finger; discharge chiefly of blood, and fetid dark matter (about six ounces); examined by introduction of the catheter as a staff, and to try what interruptions in the urethra; a little obstruction just about the curve, and on the corresponding internal side of the abscess, but it passed in tolerably easy to the bladder; a little matter and blood came through it; looks more like gonorrhœal matter than like that of the abscess, which it appears certain has not broken internally at all; as the catheter catheter is not to be felt by the minutest probing towards it through the wound; have great hopes that the urethra may be saved, and, in fact, the traces and directions of this abcess, though excited no doubt at first by disease of the urethra from obstructions there, causing inflammation, &c. I say, the direction and place of the abscess and its opening, appear to be posterior to the urethra, after it has curved under the pubis, and ascends to the bladder; and in that little space, left between the urethra and the anterior side of the rectum, a little laterally to the left of that gut, I examined thus: Catheter steadied in the bladder by an assistant, fore finger of the left hand in rectum bearing on the catheter, and then probing with the right; there is yet a good deal of thickening of the rapha, and teguments; so the probe is buried deeper, by the external state of the parts; goes in about an inch and a half; if all the swelling was reduced, suppose it might go half an inch at present; whatever further depth we have to apprehend, it goes deepest rather across the rapha, and obliquely towards the bulb of urethra; I feel by finger in the rectum, strong pulsation of branch of the pudical artery; at present do little more than cleanse the sore, and reduce the swelling. Rep. catap. op. omitt. ung. hydrarg.

28th, Last night more of the eschar broken through, forming a second hole; divided the intermediate piece of integument, so as to present a wound full an inch in diameter, and of proportionate circumference; really a large wound; no urine has yet passed, nor air from the rectum, and hopes still remain, that communication with it may be prevented, or rather not take place: for all I can do to prevent, is to keep the parts very clean, support the system, and watch the general health or action of any virus, and to act surgically as in a common abscess of a delicate part; passed the catheter again, but with much more difficulty; much blood came through it from evident obstructions at the curve of the urethra; after several attempts and some force, got it into the bladder; he has no exact stoppage of water, but feels considerable pain in micturition; it always, but not more than formerly, comes in a narrow stream; swelling, inflammation, and pain considerably abated; very little discharge of blood or matter, bottom and edges still foul and ragged; will, I think, become much larger, before we get sound parts; a good part of the parietes of the abscess being disorganized, much more than I expected, almost as if acted on by caustic; finger in the rectum, feel the catheter distinctly, but considerably siderably above, or before the rectum; so that I hope the destructive action of the abscess, though at first excited by venereal cause and obstruction in the urethra, will not extend to destroy its coats; in fact, towards it the probe scarce passes, but chiefly under the integuments; where some times however in these parts, we have disagreeable and extensive sinuses; the greatest direction of the probe is about an inch across the rapha; an aukward place, because, before it can heal, those cross layers must be divided, though apparently deep from thickening of the parts. I think it is merely beneath the integuments: such division must be aukward, as lessening the support of the loose folds of the perinæum and anus; but there is, I am sorry to say, a direction of the probe, though not easily found but in the most gentle manner, and by almost letting the probe find its way, by its own weight: thus a sinus is distinguished seemingly not of more capacity than the size of the probe, passing from the edge of the wound nearest the rapha close to the anus, and up along its left and rather anterior side, the probe being very distinctly felt by my finger in the rectum, just outside the coats, about an inch and a half in depth; not yet into the gut, though such is to be feared, or at least that it will go deeper, as the parts cleanse off, and allow the probe to pass more freely; unless on the other hand we find a kind action of nature, and restoration of parts from the bottom, so soon as all diseased action of the abscess is removed. Too soon yet to prognosticate, or look to radical means; let us hope for the best, and continue still to cleanse, palliate, and reduce the active state and effects of inflammation by poultices, &c.; keep the wound open (indeed it is pretty much so without) by lint. He is weak, reduced, and pale; no appetite, though now no fever to speak of; begin the bark, brace up and strengthen the stomach and general tone; encourage his spirits, and give moderate diet.

29th, I examined last night without the catheter; the only two parts of consequence in which the probe passes, are one about an inch under and across the rapha perinæi about an inch from the anus; it appears to be merely under the integuments, but thickened as before observed; this not being considered as likely to fill up and readily unite, especially as being loose from the parts beneath, with thickened ragged edges, or to waste away so as to produce a flat surface for healing, as it will not probably remain hollow, I have therefore to-day divided it with a scalpel, and cut off a little ragged thick angle of the inferior ferior part of the side of the wound; bled about two ounces, but no arterial saltus; I have thus an open view of the wound, for it was larger within than without. Wound begins to cleanse, looking red in nobs, having a glandular appearance, especially towards the bulb of the urethra, which is nearly exposed: but the second and more serious consideration is the opening absolutely of the nature of a sinus, though it may become more open, and easy of access now, by this dilatation, because by cutting off the rapha the sides of the wound are fallen open considerably; yet last night again passed the probe up an inch and a half on the left, and rather anterior side of the rectum; felt only separated by the coats of the intestine. This I rather think it will be necessary to divide through the gut, because the nature of those parts of gut, skin, and membranes without, are so different; therefore not likely to agglutinate by granulations, besides, in all probability from the loose folding texture of all parts here, sufficient degree of inflammation cannot probably be produced for solid healing, to obliterate and close up to the sides of the gut, without some mechanical means of irritation; the knife also acting as a more explanatory principle, procuring a complete open wound, which may be dressed and acted upon by local stimulants, as caustic, &c. and bring the whole from even the bottom of the sinus to granulate; but however, it is to be hoped, as the gut is sound and as I have now so exposed the wound, which I was more led to do, to save time, and excite healing action, by freely exposing if possible the bottom of the whole abscess, thus in a degree to stimulate the parts where the probe passes, with hopes also of that healing up; and that we may succeed without it, we are doing almost all we can well do, short of dividing the gut; at all events too soon to do that, till we see what nature a little aided now and then can do, for of course it is very desirable to avoid dividing the rectum, even for such short extent. It is a great satisfaction, that neither the urine or fæces have found their way; indeed, except by ulceration it is certainly too far behind for the urethra, which no doubt is however diseased, and the first exciting cause of the abscess. The discharge of gonorrhœal-like matter is more; passes urine easier, and had a very copious loose stool last night, so have taken off pressure on the bladder, urethra, and wound, which has diminished the appearance and sense of tension: has hitherto been very abstinent; he is weak, yet on the whole better than could be expected; his mouth is sore, and spits some-

 what; what; I thought he had naturally fetid breath, but it appears he exceeded the quantity of ung. hydrarg. when he rubbed in to repel; five grains twice a-day were ordered to the part, but he used half an ounce in a week; rather in his favour. Rep. cinchon. cataplasm ter in die. Gentle diet; he is quite easy, and bears it well.

30th, Although the general appearance of the wound is decidedly much improved, cleansed off, and beginning to look florid, with even a general sign of filling up, by rather large bulbous granulations; scarce any surrounding swelling, and no pain except when dressed; the parts lax, all the inflammatory stage and destruction of the abscess over, not the least mask of ulceration, also as a matter of great moment the sinus directed to the rectum is much more difficult to find, by no doubt a general disposition to contraction and filling up of I think healthy action, so that last night I could scarce find it, and then the probe did not pass so high;—yet against all these favourable marks there was last night a terrible disappointment; I had frequently inquired whether urine or air passed through the wound; always answered in the negative; yet to be better ascertained, desired him to keep his water till the evening dressing, to see him make it, which he did about half a pint; I suspected the evil before he had well begun, by seeing a trickling of thin fluid from the edge of the wound, which was fully confirmed whilst straining in the act of micturition by a dribbling of urine through the wound, pretty high up, above the bulb of the urethra, in the whole about half an ounce; gave very little pain and more sense of warmth. By close questioning he says he has felt to-day, when making water, a sense of trickling and heat there; he has no idea of the nature of his infirmity. This is a sad disappointment, but a reasonable event to expect, considering the internal disease of that part and the original cause, there evidently existing narrowness of the canal; passing the catheter requiring considerable trouble, the obstructions may be felt breaking down under it, a little blood and matter being forced out: so now we have decidedly fistula ex urethrâ in perinæo, as the principal object of attention; more puzzling than the suspected disease of the rectum, which apprehension has diminished. This must now be our object; we *may* hope by a good strong habit, and the promising state of the parts, although we have to conclude there is an extensive and long-existing contraction, and no doubt thickening and callosity with occasional fungus, &c. all from the original inflammation of of the urethra, to have so disorganized that passage, as to give however very poor hope of success by bougie; can we by it, as a mechanical instrument, expect so to overcome the contraction, disposition to callosity, occasional inflammation, &c. as to succeed in obliterating the diseased edges of the opening, or so to give good and effective action, as to get this fistulous opening to heal? We may hope it, and we must try it; but then should we succeed in healing it, we must expect by future advances of the disease and occasionally exciting causes, to find inflammation, abscess, and the consequent evil renewed: yet as we may hope, it is our duty to try, especially under such favourable health; even such occasional cure may by care last long, and by the continued use of bougies the internal disease may be almost obliterated, and of course permanent cure obtained. I know but of one other indication of cure more readily radical in promise, but liable to objections; I mean a free division of the parts, as in the operation of lithotomy, and taking bougies to our assistance at the same time; the direction of the wound is nearly that of the parts divided in that operation. Rep. cinchon. cataplasm, &c. restorative diet; is quite easy but weak, and giddy; the latter affection attributed to lying in bed.

31st, Had a very copious stool in the night, no fæces or urine passed through, nor last night when dressed; he then evacuated a little urine, but none through the perinæum: yet in the course of the day when he made water he felt a drop or two pass, but none came through to-day when he made water at the time of dressing; the opening at the side of the rectum much more difficult to find, and less deep; the general appearance of the wound still improves, florid, granulating, &c. but in its centre you see now pointed out by the hollow left, the trace to the opening of the urethra, or where it was, is partially closed; he spits more by the increasing effects of mercury; not against him. Rep. cinchon. Good diet; began to-day to act on the principles yesterday spoken of by the bougie, but they are so ill made, soft, and totally ill adapted, that I could not pass it further than the point of the obstruction; the least force to pass it further, upset all endeavour, although one of a moderate size; so in this I fear I am completely foiled. I have an idea of keeping in a small catheter, which though it will act only mechanically, will have this advantage, that it may be retained as long as you please, and not be withdrawn to make water; perhaps it will be for the best, as it can irritate only by its

 pressure, pressure, the other might also by its parts; besides, the patient, when he wants to make water, has such sudden call that he cannot wait; it acts at such unequal times, so would be teazing, and obliged to have the bougie withdrawn; but a catheter, by means of the stillet, may be kept till pain or pressure alone make it necessary. On this principle it would be better that all bougies of common texture, and which could be long retained, were made all as catheters; which if more firm, as at all events they ought to be, might easily be the case, and when water necessary to be made could either (if formed even of a stimulant nature), withdraw the whole, or the stillet, according to the feeling of the patient. The superiority of the flexible metallic bougie here occurred to me, being at once more pliable and stiff, but even so they would be better formed as catheters; and as there are catheters so formed, where is the use of bougie at all, as the former will answer all the purposes? And here by the bye, now I think of it, the stillet of the gum elastic catheter is flexible metal, and being large is positively equal to a bougie of the nature required; so can try that to dilate the passage first gradually, before the silver catheter is used, and of the last, or of common bougie when once I can introduce it, may I not be able to act on the principle of Mr. Homes's bougies with caustic? I think I may be able, at least in the common bougie, to insert a bit of caustic.

February 1st, A dreadful change indeed has taken place; decided mortification; how far it extends, or how far it will go, I do not know, but he appears to be in imminent danger; and all this at once from the height of expectation, to my great alarm and the greatest evil to him; though in no pain, I scarce think it possible he can live; the wound is quite black all over, but principally the edges, and surrounded with that peculiar pale redness like erysipetalous inflammation, about the size of a crown piece, taking in the whole circle of the anus, thus far therefore, mortification will go; for we never yet saw parts having taken on this inflammation of gangrene, without in their turn falling into gangrene also; and as I fear (as will be shewn by and by from proper grounds), that the affection is deep; so that if life should be preserved, that the disease will go far beyond the integuments, and of course the rectum, some of the muscles, and also perharps scrotum and urethra slough off; what a dreadful state, therefore will he be reduced to; worse than death; if he lingers, it it will be under an accumulation of distress, but if he dies from gangrene, I think it will be in about 30 hours, under the prostration and final delirium of sphacelus; can I expect a man to live on whom has advanced on a sudden such deplorable symptoms? I shall relate the occurrences, and then leave judgment to decide.

(To be continued.)

